# Psychometric Properties of the Chinese Translated Athlete Burnout Questionnaire: Evidence From Chinese Collegiate Athletes and Elite Athletes

**DOI:** 10.3389/fpsyg.2022.823400

**Published:** 2022-05-06

**Authors:** Hao Liu, Xiang Wang, Dong-Hai Wu, Yu-Duo Zou, Xiao-Bo Jiang, Zhi-Qing Gao, Ri-Hong You, Jin-Chuan Hu, Jing-Dong Liu

**Affiliations:** ^1^Department of Physical Education, Shenzhen University, Shenzhen, China; ^2^Department of Curriculum and Instruction, The Education University of Hong Kong, Tai Po, Hong Kong SAR, China; ^3^Department of Physical Education, Sun Yat-sen University, Guangzhou, China; ^4^Hong Kong Sports Institute, Sha Tin, Hong Kong SAR, China; ^5^Beijing Institute of Sport Science, Beijing, China; ^6^Shenzhen Kangning Hospital, Shenzhen, China

**Keywords:** athlete burnout, collegiate athletes, validity, reliability, measurement invariance analysis

## Abstract

The purpose of the study was to translate the athlete burnout questionnaire (ABQ) into Simplified Chinese and examine its psychometric properties in Chinese collegiate athletes and elite athletes. Firstly, the factor structure, internal consistency reliability and nomological validity of the Chinese translated ABQ was examined in a sample of Chinese collegiate athletes (*n* = 214, 58.9% females). Secondly, abovementioned psychometric properties were examined in a sample of Chinese elite athletes (*n* = 505, 52.7% females). Finally, measurement invariance of the Chinese translated ABQ was examined across the two samples. It was found that the 12-item three-correlated-factors model outperformed the one factor model and bi-factor model in collegiate athlete sample whereas the 12-item bi-factor model best represented the factor structure of the Chinese translated ABQ in elite athlete sample. Satisfactory internal consistency reliabilities of the Chinese translated ABQ were evidenced in the two samples. Nomological validity was also supported by the results of the two samples that the three subscales of the ABQ were significantly associated with its theoretically related variables. Results of multiple-group confirmatory factor analysis revealed that weak measurement invariance of the Chinese translated ABQ (three-correlated-factors model) was evidenced across the two samples. Collectively, results of this study indicated that the 12-item Chinese translated ABQ could be used for measuring burnout of Chinese collegiate and elite athletes. Significance and implication of the current study as well as recommendations for future study were discussed.

## Introduction

Athlete burnout has been recognized as a serious and increasing problem within sport because it is closely related to athletes’ negative experiences including extreme exhaustion, decreased motivation, poor training quality, underperformance, compromised physical and psychological wellbeing and quitting thoughts or behaviors (Eklund and DeFreese, 2015; Isoard-Gautheur et al., 2016; Gustafsson et al., 2017, 2018). Previous research has extensively explored factors that may contribute to athlete burnout from both qualitative and quantitative perspectives (Udry et al., 1997; Cresswell and Eklund, 2006, 2007; Chang et al., 2018; Gustafsson et al., 2018; Pacewicz et al., 2019). For example, Gould et al. (1996) found that four main factors that belonging to social/interpersonal, physical, logistical and psychological aspects contributed to their burnout. A recent meta-analysis synthesized 20 quantitative studies involving 5,501 participants and revealed that social support and relatedness displayed significant and negative relationships with burnout whereas negative social interactions was positively associated with burnout (Pacewicz et al., 2019), which provided empirical evidence for previous qualitative findings. Some other quantitative research has also revealed that personal factors such as perfectionism, performance-based self-esteem, controlled motivation, psychological needs satisfaction and frustration, perceived stress, and sport commitment were significantly associated with burnout (Schmidt and Stein, 1991; Raedeke, 1997; Goodger et al., 2007; Lonsdale and Hodge, 2011; DeFreese and Smith, 2013; Li et al., 2013; Madigan et al., 2015; Gustafsson et al., 2017). Moreover, it was found that athlete burnout was positively associated with negative affect, worry and concentration disruption whereas negatively related to positive affect, subjective vitality, enjoyment, commitment and well-being (Raedeke and Smith, 2001; Lemyre et al., 2006; Lonsdale et al., 2008; Isoard-Gautheur et al., 2010; Gerber et al., 2018). In addition, some psychological constructs were found to mediate the relationships between social and personal factors with burnout. For example, team communication, coach-athlete relationship, intrinsic motivation and satisfaction of competence and autonomy were found to mediate the influence of autonomy support from coach on athlete burnout (Isoard-Gautheur et al., 2012; Choi et al., 2020). Autonomous motivation mediated the relationship between perfectionistic strivings and burnout whereas controlled motivation mediated the relationship between perfectionistic concerns and burnout (Madigan et al., 2016). These studies contributed to our better understanding of athlete burnout and revealed important preventive and risk factors that researchers and practitioners need to pay attention to in their research and practice.

Lack of valid domain-specific measures of burnout has hampered athlete burnout research. Development of valid and reliable measures of athlete burnout will facilitate research on the topic by providing researchers valid tools to test and advance athlete burnout related theories and ensure practitioners to believe the results obtained from the measures. The results derived from valid and reliable measures will further inform practitioners about preventing and alleviating burnout symptoms of athletes. A clear and accurate definition of athlete burnout is critical for measurement development (Raedeke and Smith, 2001). At early stage, from a stress perspective, Smith (1986) described burnout as a psychological, emotional and at times physical withdrawal from a formerly pursued and enjoyable activity due to chronic stress or dissatisfaction. However, this definition highlights the stress from social contexts such as coach and administrators whereas pays little attention to the training stress (Gustafsson et al., 2017). Silva’s (1990) follow up research shed light on the influences of overtraining on athlete burnout, but non-training factors which are salient in burnout were not well addressed. However, they failed to provide satisfactory explanation on the question that why some athletes develop burnout whereas others do not. Although Smith (1986) mentioned that motivational and personality factors may play a role, he did not elaborate on how these factors contribute to the risk of burnout. From a quite different perspective, Coakley (1992) considered burnout as a symptom of the process of leaving sports, which is a product of the social organization of sport, and treated identity development and lack of control as salient influencing factors. Furthermore, Raedeke (1997) proposed the entrapment concept by integrating the commitment perspective and the ideas relevant to identity development proposed by Coakley to explain why some athletes burn out and some do not. Another important contribution made by Raedeke (1997) to athlete burnout research is that he adapted the three core burnout components proposed by Maslach and Jackson (1984) into more sport-specific components. Maslach and Jackson (1984) viewed burnout among human service providers as a psychological syndrome represented by emotional exhaustion, reduced sense of accomplishment and depersonalization. Taking sport-specific characteristics into consideration, Raedeke (1997) argued that athlete burnout could be represented by emotional and physical exhaustion associated with the intense demands of training and competing, which was supported by previous research of significant links between burnout and feelings of exhaustion (Cohn, 1990; Gould et al., 1996). Moreover, athlete burnout could be reflected by athletes’ feelings of reduced sense of accomplishment in terms their sport skills and abilities. This argument was also supported by previous findings that unmet expectations and inability to achieve athletes’ personal goals are associated with burnout (Gould et al., 1996). Furthermore, Raedeke (1997) argued that depersonalization was more related to human service providers but not suitable for athletes and proposed depersonalization could be replaced by sport devaluation, which reflects a state that athletes stop caring about those important issues such as sport and their own performance for them. Collectively, Raedeke and Smith (2001) proposed that athlete burnout could be featured by three components, namely, emotional and physical exhaustion, reduced sense of accomplishment and sport devaluation. Specifically, emotional/physical exhaustion reflects a state of emotionally and physically exhaustion deriving from the psychological and physiological demands that associated with training and competition. Sport devaluation refers to the development of a cynical attitude, negative feelings toward sport participation, and lack of interests and investment in sport. A reduced sense of accomplishment reflects a state that athletes negatively evaluating their achievement and abilities (Raedeke, 1997). Based on the definition, they developed the first sport-specific measure of athlete burnout, the Athlete Burnout Questionnaire (ABQ; Raedeke and Smith, 2001), and demonstrated that the ABQ displayed sufficient validity and reliability, which contributed significantly to the athlete burnout research (Gustafsson et al., 2017).

According to Raedeke and Smith (2001), the Athlete Burnout Questionnaire is a multidimensional instrument that includes 15 items measuring three dimensions, namely, emotional and physical exhaustion (five items), reduced sense of accomplishments (five items), and sport devaluation (five items). Since the development of the ABQ, it has been widely used in athlete burnout research among youth athletes, professional athletes and collegiate athletes from different countries (Isoard-Gautheur et al., 2012; Gabana et al., 2017; Lee et al., 2017) and has been translated into multiple languages, such as, French (Isoard-Gautheur et al., 2010), Spanish (Arce et al., 2012), German (Gerber et al., 2018), Norwegian (Lemyre et al., 2006), Portuguese (Guedes and Souza, 2016), and Swedish (Gustafsson and Skoog, 2012). Chen and Zhou (2007) translated the ABQ into Simplified Chinese and examined its psychometric properties among two samples of elite athletes from China. However, it was found that the three-correlated-factors model of the Chinese translated ABQ displayed a poor fit to the data (χ^2^ = 298.6, *df* = 88, CFI = 0.78, GFI = 0.85, NNFI = 0.73, RMSEA = 0.10) and the internal consistency reliabilities of reduced sense of accomplishment (Cronbach’s α = 0.17) and sport devaluation (Cronbach’s α = 0.69) were unsatisfactory. Although Chen and Zhou (2007) further revised the ABQ by removing two items and revising other items substantially, they did not elaborate on which items were removed and the rationale behind. More importantly, the measurement model of the revised version ABQ displayed a marginal fit to the data (χ^2^ = 89.64, *df* = 60, CFI = 0.91, GFI = 0.89, NNFI = 0.89, RMSEA = 0.07) and the loading of one item was below 0.50 (−0.23). Collectively, endeavors made by Chen and Zhou (2007) should be acknowledged but the psychometric evidences for the Simplified Chinese version ABQ by Chen and Zhou (2007) were not convincing enough to support for its application among Chinese athletes. Researchers from Taiwan translated the ABQ into traditional Chinese (Lu et al., 2006) and examined its psychometric properties among collegiate athletes from Taiwan. It is noteworthy to mention that Simplified Chinese and Traditional Chinese are two different writing systems between which the most noticeable differences are the characters. Traditional Chinese characters are made up of many linked and interrelated radicals while Simplified Chinese characters appear to be clearer and more straightforward. In other words, fewer stokes are needed to write a Simplified Chinese character compared with its Traditional Chinese counterpart. Another important difference between the two systems is that the Simplified Chinese merged characters, which means that each character in Simplified Chinese maps to one or more Traditional Chinese characters. There are many terms and phrases that differ in meaning between Traditional and Simplified Chinese. Therefore, it is easier for people who use Traditional Chinese to understand (although not absolutely) Simplified Chinese, but it would be more difficult for people who use Simplified Chinese to understand Traditional Chinese. Traditional Chinese is commonly used in some Chinese societies such as Taiwan, Hong Kong SAR, and Macau, whereas Simplified Chinese is commonly used in other Chinese societies such as mainland China, Singapore and some regions in Malaysia. This is one of the reasons that, for some adapted psychological measures, there are usually both Traditional Chinese version and Simplified Chinese version meanwhile. Although the Traditional Chinese version of the ABQ demonstrated satisfactory validity and reliability among Taiwan collegiate athletes (Lu et al., 2006), it may be not applicable for collegiate or elite athletes from mainland China because they may have difficulties in recognizing the Traditional Chinese characters which, in turn, may influence theirs understanding of the sentences correctly. Therefore, it is imperative to validate a Simplified Chinese version of ABQ, which will facilitate future research on burnout of athletes who use Simplified Chinese and cross-cultural studies involving those Chinese athletes.

Regarding the factor structure of the ABQ, the theoretically assumed three-correlated-factors model has been extensively examined and consistently replicated in previous research (Raedeke and Smith, 2001; Lu et al., 2006; Gerber et al., 2018). The three-correlated-factors model was found better than one-factor model consistently and inter-factor correlations of the three-correlated-factors model ranged from 0.30 to 0.71 (Raedeke and Smith, 2001). Meanwhile, a second-order model of ABQ was proposed and examined in which the three factors were considered as first-order factors and a global burnout was treated as a second-order factor (Isoard-Gautheur et al., 2010; Gerber et al., 2018) in explorations of the correlations between athlete burnout and other variables (Cresswell and Eklund, 2005; DeFreese and Smith, 2013; De Francisco et al., 2016; Ai-Yaaribi and Kavussanu, 2017; Gerber et al., 2018). However, the correlations between specific factors and the higher-order factor (global burnout) varied a lot ranging from 0.4 to 0.97, which raises an important question that whether the three symptoms could be grouped under a common factor (Shirom and Melamed, 2006; Gerber et al., 2018). Another important and unwell addressed question regarding the factor structure of the ABQ is that should the subscale scores or the total score of ABQ be computed and used (Reise et al., 2013)? Interpreting specific factors as meaningful constructs or simply computing a total score based on no empirical evidence may be misleading (Di et al., 2021). If the true underlying structure is multidimensional, the use of total score may overlook the unique contribution of specific factors. Similarly, if the true underlying structure is unidimensional, the use of subscale scores may be redundant. Bi-factor model has been recommended as an important approach for testing of multidimensional constructs because it allows researchers to examine a single common factor that represents a multidimensional construct and meanwhile acknowledge the uniqueness of the specific facets that comprise it (Reise et al., 2007; Chen et al., 2012; Stefansson et al., 2016). In other words, in the bi-factor model, the general factor reflects the conceptually broad construct that an instrument was designed to measure, and the specific factors reflect the more conceptually narrow sub-dimensional constructs (Reise, 2012; Stefansson et al., 2016). It can simultaneously estimate the different role of general factor and specific factors (Reise et al., 2016; Rodriguez et al., 2016). If specific factors in a bi-factor model explained little variance in the scores, an overall score should be computed. Otherwise, subscale scores should be computed as well (Raykos et al., 2019; Di et al., 2021). To the best of our knowledge, no previous research has examined the factor structure of the ABQ using a bi-factor model approach and provided convincing evidence for the application of the ABQ in both practice and research. Therefore, further investigation on the factor structure of the ABQ will add new information to the athlete burnout research literature and inform practitioners and researchers on the usage of the ABQ (either subscale scores or general score or both) in their practice.

Measurement invariance assesses the psychometric equivalence of a construct across groups or measurement occasions, which is one of the key psychometric properties of instruments and the prerequisite of group comparisons (Meredith, 1993). A widely used measurement invariance approach includes four-step tests of configural invariance (equivalence of model form), metric invariance (equivalence of factor loadings), scalar invariance (equivalence of item intercepts), and residual invariance (equivalence of items’ residuals or unique variances) (Meredith, 1993). Evidence of configural invariance is the prerequisite of the latter three tests and evidence of metric, scalar and residual invariance were considered as week, strong and strict invariance, respectively. Measurement invariance has been widely recognized and examined in instrument validation and adaptation studies in various fields (Putnick and Bornstein, 2016). However, to our best of knowledge, the measurement invariance evidence of ABQ was still scarce. The only reported measurement invariance evidence for the ABQ was from young Brazilian athletes by Guedes and Souza (2016). In their study, they found that the three-correlated-factors measurement model of the ABQ was invariant (factor loading, factor variance/covariance, and residual) across gender and age groups (≤14 years, 15–16 years, and 17 years). No previous research has examined the measurement invariance of either the Traditional Chinese version or the Simplified Chinese version of ABQ yet.

Collectively, to shed light on the abovementioned limitations, the purpose of the present study was to translate the ABQ into Simplified Chinese adopting standard translation-back translation strategy and further examine its psychometric properties in collegiate athletes and elite athletes from China. Specifically, factor structure of the ABQ was examined by comparing the three measurement models (M1, one-factor model; M2, three-correlated-factors model; M3, bi-factor model). Internal consistency reliability was examined using Cronbach’s alpha (α) and coefficient Omega (ω). Moreover, nomological validity of the Chinese Translated ABQ was examined by evaluating the correlations between ABQ subscales with its theoretically related variables. Finally, measurement invariance of the Simplified Chinese version ABQ was examined across the two samples.

## Materials and Methods

### Participants and Procedure

#### Collegiate Athlete Sample

A total of 226 collegiate athletes agreed to participate in this study by completing a set of questionnaires measuring athlete burnout, worry and concentration disruption, as well as subjective vitality. Excluding invalid and incomplete data, data of 214 participants (88 females and 126 males; age: *M* = 21.42, SD = 1.24) were identified as valid and used for data analysis. Collegiate athletes were from seven sports including basketball, table tennis, swimming, fencing, track and field, football and volleyball with an average of 6.4 years (SD = 3.78) engaging in their sports. Collegiate athletes are those who study at universities and meanwhile compete at provincial and national level games (sports competitions organized for university students) representing their own universities at the moment of data collection.

#### Elite Athlete Sample

A total of 515 elite athletes agreed to participate in this study by completing a set of questionnaires measuring athlete burnout, positive affect, negative affect, concentration distraction and subjective vitality. Excluding invalid and incomplete data, data of 505 participants (266 females and 239 males; age: *M* = 18.47, SD = 4.01) were identified as valid and used for data analysis. Elite athletes were from fourteen sports including basketball, football, volleyball, track and field, table tennis, swimming, badminton, boxing, taekwondo, martial art, baseball, judo, weight-lifting and hockey with an average of 5.72 years (SD = 3.36) engaging in their sports. Elite athletes are those who are full time athletes and compete at national and international level games at the moment of data collection.

#### Translation and Back-Translation

The translation and back-translation procedure were used in this study (Guillemin et al., 1993). Specifically, two translators translated the ABQ from English into Chinese independently. Consensus on the Chinese translated ABQ was achieved by the two translators based on their discussion. Further, the Chinese translated ABQ was translated back into English by another two translators independently. A comparison of the back-translated English version with the original English version found that the meaning of the items was identical. Finally, 15 collegiate athletes and 10 elite athletes were invited to complete the Chinese translated ABQ. They reported that the instructions and items in the Chinese translated ABQ were clear and easy to understand.

#### Data Collection

Coaches and teachers were contacted to invite their athletes to participate in this study. For collegiate athletes, researchers went to their training venues personally and instructed them to complete the questionnaires onsite without the presence of their coaches and teachers. Informed consent was distributed and obtained before data collection and only participants who returned their consent form were asked to complete the questionnaires. Data were collected between March and June, 2018. For elite athletes, they finished the online survey because of the COVID-19 Lockdown. Informed consent was obtained before data collection and only those who returned their consent were sent the web-links of online survey through their coaches. The instruction of survey was clearly stated at the beginning of the survey. Data were collected between September and November, 2020. For all participants who were younger than 18 years old, their coaches were invited to be as their acting agent and consent form from their coaches were obtained before data collection. All participants took part in the study voluntarily.

### Measures

#### Athlete Burnout

Athlete Burnout Questionnaire (Raedeke and Smith, 2001) was used to measure participants’ burnout. The ABQ includes 15 items measuring emotional/physical exhaustion (five items), reduced sense of accomplishments (five items) and sport devaluation (five items). Responses were measured on a 5-point Likert scale ranging from 1 (never) to 5 (always). Previous research has consistently revealed that the ABQ had satisfactory validity and reliability (Raedeke and Smith, 2001; Arce et al., 2012; Guedes and Souza, 2016; Gerber et al., 2018).

#### Worry and Concentration Disruption

Eight items from Chinese version of the Sport Anxiety Scale-2 (SAS-2; Smith et al., 2006) were used to measure participants’ worry (four items; example item such as it is hard to concentrate on the game) and concentration disruption (four items; example item such as I worry that I won’t play well) (Wang, 2021). Responses were measured on a 4-point Likert scale ranging from 1 (never) to 4 (always). Previous research revealed that the SAS-2 displayed satisfactory validity and reliability among Chinese elite athletes (Wang, 2021). Internal consistency reliability of the concentration disruption and worry subscales in previous research (worry: 0.756, concentration disruption, 0.896, Wang, 2021) and in the present study were satisfactory (collegiate athlete sample: worry, 0.753 and concentration disruption, 0.807; elite athlete sample: concentration disruption, 0.895).

#### Subjective Vitality

A Chinese version of Subjective Vitality Scale (SVS; Ryan and Frederick, 1997) was employed to measure participants’ subjective vitality, which has shown good psychometric properties (Liu and Chung, 2019). The scale includes five items and responses were measured on a 7-point Likert scale ranging from 1 (not at all) to 7 (very true). Previous research revealed that the simplified Chinese version of the SVS displayed satisfactory validity and reliability among Chinese elite athletes (Wang, 2021) and collegiate athletes (You et al., 2017). Internal consistency reliability of the scale in previous research (collegiate athlete sample: 0.89; elite athlete sample: 0.955) and in the present study were satisfactory (collegiate athlete sample: 0.870; elite athlete sample: 0.954).

#### Positive and Negative Affect

A Chinese version of the International Positive and Negative Affect Schedule-Short Form (I-PANAS-SF) was used to measure participants’ positive and negative affect, which has shown good psychometric properties (Liu et al., 2020). The scale includes nine items with four items measuring positive affect and five items measuring negative affect. Responses were measured on a 5-point Likert scale ranging from 1 (never) to 5 (always). Previous research revealed that the Chinese version of the I-PANAS-SF displayed satisfactory validity and reliability among Chinese elite athletes (Wang, 2021). Internal consistency reliability of the positive affect and negative affect subscales in previous research were 0.82 and 0.758 and in the present study were 0.88 and 0.755, respectively.

### Data Analysis

SPSS and AMOS (Version 23.0, Armonk, NY, United States: IBM Corp.) were used for data analysis. Firstly, given that collective evidences suggested that the three-correlated-factors model of the ABQ had been widely replicated, in this study, we chose to examine the factor structure of the ABQ using CFA rather than exploratory factor analysis (EFA). Specifically, three measurement models of the ABQ were examined and compared among collegiate athletes and elite athletes, respectively. In the first model, all items freely loaded on one general latent factor (M1: one-factor model). In the second model, the three factors were correlated with each other (M2: three-correlated-factors model). In the third model, items regressed on their intended factors and meanwhile all items freely loaded on a global factor with the three factors uncorrelated with each other (M3: bi-factor model). Moreover, internal consistency reliability of each subscale was evaluated using Cronbach’s α and coefficient Omega (ω). Furthermore, to be consistent with previous ABQ research, nomological validity of the ABQ was evaluated by examining the correlations of the subscale scores of the ABQ with its theoretically related variables. If the findings of the present study were consistent with previous results, it would be considered that the nomological validity of the ABQ was supported (Raedeke and Smith, 2001). For collegiate athletes, correlations between athlete burnout and worry, concentration disruption as well as subjective vitality were examined. For elite athletes, correlations between athlete burnout and concentration disruption, negative affect, positive affect and subjective vitality were examined. Finally, multiple-group CFA was performed to examine the measurement invariance of the ABQ (commonly shared measurement model) across collegiate athletes and elite athletes. For measurement invariance analysis, four models were evaluated: configural (M1), metric in-variance (M2: weak invariance), scalar invariance (M3: strong invariance) and residual invariance (M4: strict invariance).

Multi-fit indices were used to evaluate the adequacy of the model fit to the data, including the Chi-square value, comparative fit index (CFI), Tucker–Lewis index (TLI), root mean square error of approximation (RMSEA) accompanied by its 90% confidence interval (CI) and standardized root mean square residual (SRMR). Specifically, a threshold of >0.90 for the CFI and TLI, of close to (or less than) 0.08 for the SRMR and of up to 0.08 for the RMSEA represented an acceptable fit. CFI and TLI values exceeding 0.95, and SRMR and RMSEA values close to (or less than) 0.08 and 0.06, respectively, represented a good fit (Hu and Bentler, 1999). Differences in model fit were confirmed with a chi-square difference test, which was further supplemented by comparing the Akaike information criteria (AIC) and the Bayesian information criterion (BIC) values with lower AIC and BIC values indicating a better fit. For bi-factor model, various statistical indices relevant to evaluation of bi-factor models were used including the Percent of uncontaminated correlation (PUC), Explained common variance (ECV), Omega (ω), and Omega H (ωH), which were computed using Bi-factor Indices Calculator (Dueber, 2017). The PUC represents the percentage of covariance terms that only reflect variance from the general factor and the ECV represents the proportion of all common variance explained by the general factor. Omega is a model-based estimate of internal reliability of multidimensional composite whereas Omega H represents the percentage of systematic variance in unit-weighted total score that can be attributed to the individual differences on the general factor. Reise et al. (2013) argued that when PUC value was lower than 0.80, general ECV value greater than 0.60 and Omega H larger than 0.70, it would indicate the presence of multidimensionality but which was not severely enough to disqualify the interpretation of the instrument as unidimensional. For measurement invariance evaluation, as the Chi-square difference test depends on sample size, the differences in the descriptive fit indices (ΔCFI, ΔRMSEA, and ΔSRMR) were used in model comparisons in this study. According to Chen (2007), when testing metric invariance, non-invariance is indicated by a change of ≥ 0.010 in the CFI supplemented by a change of ≥ 0.015 in the RMSEA or ≥ 0.030 in the SRMR; when testing scalar or residual invariance, non-invariance is indicated by a change of ≥ 0.010 in the CFI supplemented by a change of ≥ 0.015 in the RMSEA or ≥ 0.010 in the SRMR.

## Results

### Results of Collegiate Athletes

#### Factor Structure and Internal Consistency Reliability

Model fit indices of the three models were presented in Table 1. It was found that the model fit of the three 15-item models were below the acceptable criterion. Examination of the factor loadings (three-correlated-factors model) revealed that two items of reduced sense of accomplishment (item 1: 0.28; item 14: 0.42) and 1 item of sport devaluation (item 3: 0.40) performed poorly with factor loadings lower than 0.50 (Hair et al., 2014). After removing the three items, the model fit of the 12-item one-factor model and the bi-factor model were improved but still inadequate. The three-correlated-factors model demonstrated acceptable and much improved model fit to the data, χ^2^ = 107.035, *df* = 51, CFI = 0.941, TLI = 0.923, SRMR = 0.060, RMSEA = 0.072 (90% CI = 0.053–0.091). The standardized factor loadings ranged from 0.549 to 0.759 (Table 2). The inter-factor correlations of the three-correlated-factors model ranged from 0.62 to 0.77 (*r* between reduced sport achievement and emotional and physical exhaustion = 0.62; *r* between emotional and physical exhaustion and sport devaluation = 0.77; *r* between reduced sport achievement and sport devaluation = 0.73). The three-correlated-factors model outperformed the one-factor model and the bi-factor model based on the difference tests together with the changes in AIC and BIC values (see Table 1).

**TABLE 1:** Model fit indices of measurement models.

Sample	Model	χ^2^	df	CFI	TLI	RMSEA (90% CI)	SRMR	AIC	BIC	Comparison	Δχ^2^ (Δdf)
Collegiate athletes	M1 (15 items)	307.427	90	0.797	0.763	0.106 (0.094−0.120)	0.083	367.427	468.406		
(*n* = 214)	M2 (15 items)	199.992	87	0.894	0.873	0.072 (0.064−0.092)	0.072	265.992	377.069		
	M3 (15 items)	294.267	79	0.799	0.733	0.113 (0.099−0.127)	0.127	376.267	514.272		
	M1 (12 items)	208.124	54	0.837	0.801	0.116 (0.099−0.133)	0.079	256.124	336.908		
	M2 (12 items)	107.035	51	0.941	0.923	0.072 (0.053−0.092)	0.060	161.035	251.916	M2 vs. M1	−101.089 (3)***
	M3 (12 items)	153.869	46	0.886	0.836	0.105 (0.087−0.123)	0.061	217.869	325.580	M3 vs. M2	46.834 (5)***
Elite athletes	M1 (15 items)	521.888	90	0.893	0.876	0.098 (0.090−0.106)	0.059	581.888	708.625		
(*n* = 505)	M2 (15 items)	399.905	87	0.923	0.907	0.084 (0.076−0.093)	0.055	465.905	605.316		
	M3 (15 items)	467.095	79	0.904	0.873	0.099 (0.090−0.107)	0.128	549.095	722.302		
	M1 (12 items)	354.123	54	0.919	0.901	0.105 (0.095−0.116)	0.051	402.123	503.512		
	M2 (12 items)	234.350	51	0.951	0.936	0.084 (0.074−0.096)	0.044	288.350	402.413	M2 vs. M1	−119.773 (3)***
	M3 (12 items)	171.379	46	0.966	0.952	0.074 (0.062−0.085)	0.039	235.379	370.565	M3 vs. M2	−62.971 (5)***

M1, one-factor model; M2, three-correlated-factors model; M3, bi-factor model; CFI, comparative fit index; RMSEA, root mean square error of approximation; SRMR, standardized root mean square residual; 90% CI, 90% confidence interval; AIC, Akaike information criteria; BIC, Bayesian information criterion; Δχ^2^, changes in Chi-square; Δdf, changes in degree of freedom. ***p < 0.001.

**TABLE 2:** Descriptive statistics, factor loadings and internal consistency reliabilities of the Chinese translated ABQ (12-item three-correlated-factors CFA).

Subscales and items	Collegiate athletes (*n* = 214)								Athletes (*n* = 505)							
	*M*	SD	EPE	SDE	RSA	ω	AVE	α	*M*	SD	EPE	SDE	RSA	ω	AVE	α
EPE						0.869	0.459	0.808						0.889	0.538	0.818
Item 2	2.70	0.830	0.638						2.76	1.259	0.500					
Item 4	2.55	0.957	0.601						2.59	1.226	0.619					
Item 8	2.14	0.994	0.697						2.01	1.181	0.835					
Item 10	2.66	1.075	0.705						2.19	1.236	0.815					
Item 12	2.25	1.007	0.736						2.02	1.186	0.834					
SDE						0.861	0.488	0.780						0.927	0.634	0.755
Item 6	2.56	1.268		0.748					1.99	1.145		0.805				
Item 9	2.86	1.067		0.729					1.96	1.148		0.875				
Item 11	2.66	1.159		0.748					1.87	1.132		0.831				
Item 15	2.27	1.158		0.549					1.90	1.163		0.655				
RSA						0.859	0.520	0.762						0.898	0.554	0.787
Item 5	2.86	0.983			0.654				2.50	1.200			0.647			
Item 7	2.76	1.054			0.745				2.37	1.269			0.783			
Item 13	2.60	1.019			0.759				2.24	1.267			0.794			

*EPE, emotional and physical exhaustion; SDE, sport devaluation; RSA, reduced sport achievement; M, mean; SD, standardized deviation; AVE, average variance extracted.*

Although the model fit of the bi-factor model was inferior, additional indicators for the bi-factor model was also examined. It was found that the ECV was 0.584, which indicated that the general ABQ factor contributed to 58.4% of the common variance whereas the three specific factors accounted for 41.6% of the common variance. Meanwhile, PUC was 0.712 and omega H was 0.791, which indicated the multidimensional nature of the ABQ (Reise et al., 2013). In other words, it would be best to apply the ABQ among collegiate athletes at subscale level rather than at the general or total score level. Cronbach’s α coefficients of three subscales ranged 0.762–0.808 and coefficient Omega of the three subscales ranged from 0.859 to 0.869. Average variance extracted (AVE) values of the three subscales ranged from 0.459 to 0.520 (see Table 2). These results suggested that the 12-item ABQ had satisfactory reliability.

#### Nomological Validity

Correlations of the scores derived from the 12-item Chinese translated ABQ with its theoretically related variables suggested that the subscale scores of the ABQ were positively and low to moderately associated with worry and concentration disruption and negatively and moderately correlated with subjective vitality (see Table 3). These results were consistent with previous studies and provided support for the nomological validity of the Chinese translated ABQ in collegiate athletes.

**TABLE 3:** Correlations of subscale scores and total score of the Chinese ABQ with its theoretically related variables.

Burnout	Collegiate athletes (*n* = 214)			Athletes (*n* = 505)			
	Worry	Disruption	Subjective vitality	Disruption	Negative affect	Positive affect	Subjective vitality
RSA	0.396	0.492	−0.413	0.542	0.513	−0.384	−0.415
EPE	0.235	0.435	−0.421	0.548	0.503	−0.433	−0.472
SDE	0.296	0.520	−0.469	0.577	0.456	−0.466	−0.492
Burnout	−	−	−	0.606	0.536	−0.465	−0.500
α	0.807	0.753	0.870	0.895	0.818	0.755	0.954

*EPE, emotional and physical exhaustion; SDE, sport devaluation; RSA, reduced sport achievement; α, Cronbach’s α; all coefficients were significant at 0.01 level.*

### Results of Elite Athletes

#### Factor Structure and Internal Consistency Reliability

Model fit indices of the three 15-item models were presented in Table 1. The one-factor model and the bi-factor model displayed a poor fit to the data while the three-correlated-factors model demonstrated an acceptable fit to the data. However, examination of the factor loadings (three-correlated-factors model) revealed similar results of collegiate athletes that two items of reduced sense of accomplishment (item 1: 0.44; item 14: 0.36) and 1 item of sport devaluation (item 3: 0.34) performed poorly with factor loadings lower than 0.50 (Hair et al., 2014). These results suggested that the three items were problematic among elite athlete sample too. After removing the three items, all three 12-item models displayed an acceptable and much improved model fit to the data. The standardized factor loadings ranged from 0.50 to 0.88 (Table 2). The inter-factor correlations of the three-correlated-factors model ranged from 0.88 to 0.93 (*r* between reduced sport achievement and emotional and physical exhaustion = 0.93; *r* between emotional and physical exhaustion and sport devaluation = 0.88; *r* between reduced sport achievement and sport devaluation = 0.90). Model comparison revealed that the 12-item bi-factor model outperformed the other two models based on the difference tests together with the changes in AIC and BIC values (see Table 1). Further examination of the additional indicators for the bi-factor model revealed that the ECV was 0.747, which indicated the general ABQ factor explained about 74.7% of the common variance extracted with about 25.3% of the common variance spread across the three specific factors. Meanwhile, PUC was 0.712 and the omega H was 0.912, which indicated the presence of multidimensionality but not severely enough to disqualify the interpretation of the unidimensional of the ABQ construct (Reise et al., 2013). In other words, it is meaningful to consider both specific factors and general factor of the ABQ in practice and research. Cronbach’s α coefficients of three subscales ranged 0.755–0.818, coefficient Omega of the three subscales ranged from 0.889 to 0.927 and coefficient Omega of the total scale was 0.962. These results suggested that the Chinese translated ABQ had satisfactory internal consistency reliability. Average variance extracted (AVE) values of the three subscales ranged from 0.538 to 0.634 (see Table 2).

#### Nomological Validity

Correlations of the scores derived from the 12-item Chinese translated ABQ with its theoretically related variables suggested that the total score and subscale scores of the ABQ were positively and moderately associated with concentration disruption and negative affect and negatively and moderately correlated with positive affect and subjective vitality (see Table 3). These results were consistent with previous studies and provided support for the nomological validity of the Chinese translated ABQ in elite athletes.

### Invariance Analysis (Collegiate Athletes and Athletes)

Given the bi-factor model was found inferior among collegiate athletes and the three-correlated-factors model were evidenced among both collegiate and elite athlete samples, measurement invariance of the three-correlated-factors model across the two samples was conducted. Invariance constraints across the two samples were progressively added to the 12-item three-correlated-factors ABQ measurement model. Table 4 presents the goodness-of-fit indices for the independent and invariance models. All of the models displayed acceptable fit to the data except that of measurement residual model. Results of comparison between the more and less constrained models (M2 vs. M1; M3 vs. M2; and M4 vs. M3) across samples showed significant changes in model fit (as measured by ΔCFI, ΔRMSEA, and ΔSRMR) that exceeded the recommended cut-off values except for the comparison between M2 and M1. These results suggested that only weak invariance of the 12-item three-correlated-factors model of the Chinese translated ABQ across different samples was evidenced.

**TABLE 4:** Model fit indices of invariance analysis.

Model	χ^2^	df	CFI	TLI	RMSEA (90% CI)	SRMR	Model comparison	ΔCFI	ΔRMSEA	ΔSRMR
There-correlated-factors CFA (*n* = 214)	107.035	51	0.941	0.923	0.072 (0.053−0.092)	0.060	−	−	−	−
Three-correlated-factors CFA (*n* = 505)	234.352	51	0.952	0.936	0.084 (0.074−0.096)	0.044	−	−	−	−
M1: configural	342.237	102	0.948	0.933	0.057 (0.051−0.064)	0.044	−	−	−	−
M2: metric	350.754	111	0.949	0.939	0.055 (0.048−0.061)	0.044	M2 vs. M1	0.001	0.002	0.000
M3: scalar	504.007	123	0.918	0.912	0.066 (0.060−0.072)	0.045	M3 vs. M2	0.031	0.011	0.001
M4: residual	688.821	141	0.890	0.882	0.068 (0.036−0.050)	0.055	M4 vs. M3	0.028	0.002	0.010

*CFI, comparative fit index; RMSEA, root mean square error of approximation; SRMR, standardized root mean square residual; 90% CI, 90% confidence interval; ΔCFI, changes in CFI; ΔRMSEA, changes in RMSEA; ΔSRMR, changes in SRMR.*

## Discussion

In the past 30 years, athlete burnout has gradually become one of the most popular research topics in the field of sports psychology. Researchers have invested substantial time and resources into understanding of athlete burnout and its antecedent and consequent factors (Goodger et al., 2007; Gustafsson et al., 2017; Madigan et al., 2019; Pacewicz et al., 2019). Monitor of athlete burnout is an important component of burnout prevention work (Madigan et al., 2019). Currently, burnout is typically measured using self-report questionnaires, among which the ABQ has been the most widely used tool in previous athlete burnout research and practice. The current study aimed to translate the ABQ into Simplified Chinese and examine its psychometric properties in a collegiate athlete sample and an elite athlete sample from China. Results suggested that the 12-item Simplified Chinese translated ABQ displayed satisfactory reliability and validity and could be used to measure athlete burnout of both collegiate and elite athletes from China.

Bi-factor models have been widely used to examine the multidimensional structure of instruments in previous psychometric validation studies (Di et al., 2021). To the best of our knowledge, this is the first study to use the bi-factor model to examine the factor structure of the ABQ. Results from collegiate athlete sample suggested that the three-correlated-factors model best represented the factor structure of the ABQ over the one-factor model and bi-factor model, which provided support for the multidimensional nature of the ABQ. Poor fit of the one-factor model and bi-factor model suggests that the construct of the ABQ among collegiate athletes may be conceptualized as having three specific factors that share low level overlap. Although the bi-factor model was found inferior among collegiate athletes, additional indictors generated from bi-factor model provided informative suggestion that the ABQ should be applied at subscale level rather than global level among collegiate athletes. In contrast, results from elite athlete sample suggested that the bi-factor model best represented the factor structure of the ABQ over the one-factor model and the three-correlated-factors model. Significant better fit of the bi-factor model suggests that, rather than being strictly unidimensional or adhering strictly to a multidimensional structure, the construct of the ABQ may be conceptualized as having three specific dimensions that share substantial and meaningful overlap. The bi-factor model among elite athlete sample confirmed that a reliable general athlete burnout factor underlies of the ABQ items, regardless of their specific factors origin. Additional bi-factor model indicators further suggested that the ABQ should be applied at both global level and subscale level among elite athletes. In other words, it is reasonable to compute both subscale scores and global score of the ABQ in research and practice because both of which are meaningful. It was interesting to find out that the factor structure of the ABQ between collegiate athlete sample and elite athlete sample was different. One possible reason might due to the fact that the three specific factors of the ABQ associated with each other in a much different pattern between the two samples, in which the inter-factor correlations among collegiate athletes (0.62–0.77) were much lower than that among elite athletes (0.88–0.93). More importantly, the inter-factor correlations of collegiate athlete data were somewhat discrepant with all factors were not correlated equally strongly with each other pairwise. Although the general factor in bi-factor model accounted for whatever correlations observed between factors, when the factor correlations were not equal or similar, the general factor would not be able to equally account for the correlations between specific factors (Morgan et al., 2015). In other words, in such a case, the general factor was not really functioning as a general factor but rather as a general factor for a subset of specific factors. In contrast, the correlated factors models allow the strength of inter-factor correlations to vary. Therefore, for collegiate athletes, the three-correlated-factors model outperformed the bi-factor model. Another reason might due to the fact that the selected fit indices for model comparisons in this study were sample size sensitive (Morgan et al., 2015). To answer the argument that the bi-factor model always result in better model fit, Morgan et al. (2015) conducted a simulation research, in which they demonstrated when samples were obtained from a true multiple correlated factors structure, the fit indices would be more likely overall to identify the correlated factors solution as the best fitting and when samples were obtained from a true multidimensional bi-factor structure, the fit indices would be more likely to identify the bi-factor solution as the best fitting (Morgan et al., 2015; Di et al., 2021). These findings provided support for the rationale of model comparisons in current study, which further warrant our results. However, the likelihood of abovementioned conclusion is sensitive to the sample size. For example, Morgan et al. (2015) reported that when the true underlying model was a bi-factor model, the fit indices tended to select the bi-factor model over other models in lower likelihood (83–85%) with sample sizes of 200 than that (100%) with sample sizes of 800. When the true underlying model was the multiple correlated factors model, the fit indices tended to select the bi-factor model over other models in lower likelihood (68–70%) with sample sizes of 200 than that (99%) with sample sizes of 800. In this study, the sample size of collegiate athletes is 214, which is smaller than that of the elite athletes (*n* = 505) and close to the sample size in Morgan et al. (2015)’ simulation research. So, researchers are encouraged to further examine the bi-factor model of the ABQ among collegiate athletes by enlarging the sample size.

Results from both collegiate athlete sample and elite athlete sample consistently revealed that three items (item 1, 3, and 14) were problematic with factor loadings being lower than 0.50. Two problematic items from reduced sense of accomplishment (item 1: I have accomplished many worthwhile things in sports; item 14: I was successful in sports) are the only two reversed scoring items in ABQ. This result indicated that reversed scoring items may be not suitable for assessing burnout of Chinese athletes in the current study. Moreover, in comparison with other items related to the reduced sense of accomplishment, the expression of item 1 was somehow abstract and ambiguous whereas other items expressed clear and concrete contents that related to sport performance, ability and achievement in their sports. This ambiguity and difference in contents may be one of the reasons that item 1 performed poorly in the current sample. In addition, the results may also reflect some cultural differences. Compared with athletes from other cultures, Chinese athletes may tend to focus more on temporary and concrete reference when evaluating their accomplishment rather than to comprehend and think about the long-term and potential achievements and values that derive from their daily training and sport participation. Poor performance of the item 3 (The effort I spend in sport would be better spent doing other things) from sport devaluation may be because that the item measures athletes’ subjective feelings toward sport without clear and specific reference. Other items from sport devaluation (item 6, 9, and 11) were organized by comparing specific contents with other reference or conditions such as comparing current ability or concerns with that in the past. Future researchers may consider to develop more candidate items that taking characteristics of Chinese culture into consideration to advance the psychometric properties of the Chinese version ABQ.

Nomological validity of the ABQ was supported by finding expected positive and negative relationships with theoretical closely related variables within the nomological network. The three subscales of the ABQ were significantly and positively associated with maladaptive variables including worry, concentration disruption and negative affect and significantly and negatively associated with adaptive variables including subjective vitality and positive affect. These results were consistent with previous findings. For example, it was found that the three subscales of ABQ positively associated with worry (*rs* = 0.20–0.46), concentration disruption (*rs* = 0.19–0.40) and negative affect (*rs* = 0.22–0.31), and negatively associated with subjective well-being (*rs* = −0.30 to −0.42) (Raedeke and Smith, 2001; Lonsdale et al., 2008; DeFreese and Smith, 2013). As discussed in previous section, when the ABQ was applied among collegiate athletes, subscale scores should be computed whereas when it was applied among elite athletes, both subscale scores and total score of the ABQ should be computed. Therefore, in addition to the correlations between abovementioned variables and subscale scores of the ABQ, we also examined their relationships with total score of the ABQ among elite athlete sample. It was found that the ABQ total score displayed positive and moderate associations with concentration disruption and negative affect and negative and moderate correlations with positive affect and subjective vitality, respectively. These results are consistent with previous findings regarding the relationships between the total score of the ABQ and related variables although the supporting evidence for the use of the total score of the ABQ was absent in those studies (Lonsdale et al., 2008; DeFreese and Smith, 2013).

Cronbach’s alpha of the three subscales were also found satisfactory with all values were above 0.70 (0.705–0.818), which were comparative to that reported in previous research (Raedeke and Smith, 2001; Cresswell and Eklund, 2005; DeFreese and Smith, 2013; Olsson et al., 2021). Moreover, coefficient omega was computed in this study which ensured the satisfactory internal reliability of the ABQ subscales, which ranged from 0.856 to 0.927. For elite athletes, given the demonstration of the multidimensional bi-factor model of the ABQ and proven the meaningfulness of its global burnout score, the coefficient omega was computed (0.962) for the global burnout construct, which provided support for the internal reliability of the ABQ at a global level. Although previous research has reported the internal reliability of the ABQ at global scale level was satisfactory (DeFreese and Smith, 2013; Gabana et al., 2017; Olsson et al., 2021), this is the first study that reported the indicator of ABQ at the general factor level with justified rationale.

This is the first study that examines the measurement invariance of Chinese version ABQ. Results of multiple-group CFA revealed that weak invariance of the 12-item three-correlated-factors ABQ measurement model across collegiate athletes and elite athletes was evidenced. Weak invariance evaluates whether the change in each item score corresponds to the change in the factor score across groups. Evidence of weak invariance suggests respondents interpreting the items in similar way across groups (Horn and Mcardle, 1992). However, strong and strict invariance was not achieved in this study, which means that direct comparisons on the means scores obtained from ABQ between collegiate athletes and elite athletes should be cautioned because the two population may respond to the questionnaire differently and there maybe also some difference from measurement artifact.

Although the present study provided initial psychometric evidence for the Simplified Chinese ABQ and made significant contribution to the ABQ research literature, several limitations should be noted. First, convenience sampling was employed in this study, which may limit the generalizability of the findings obtained from this study. A larger sample size using stratified sampling approach is encouraged in future research especially for the collegiate athletes. Second, previous research has provided support for the gender invariance of the ABQ among young Brazilian athletes (Guedes and Souza, 2016). Future researchers may consider to further examine whether the Simplified Chinese ABQ measurement model would be invariant across male and female athletes from China. Third, test-retest reliability was not examined in this study and future research is encouraged to shed light on this research question. Finally, although both collegiate athletes and elite athletes were included in this study, the sample size of collegiate athletes was relatively small. Researchers are encouraged to enlarge the sample size to further examine the application of the Simplified Chinese ABQ among collegiate athletes. Instrument validation is a continuous process that involves multiple steps. More studies are needed to accumulate further evidence on the accuracy and usefulness of the 12-item Simplified Chinese ABQ.

## Conclusion

Collectively, the current study provided initial support for the psychometric properties of the Simplified Chinese version of ABQ among collegiate athletes and elite athletes from China. It could be used to measure the burnout of Chinese collegiate athletes and elite athletes using Simplified Chinese. Practically, subscale scores of the ABQ should be used for Chinese collegiate athletes whereas both subscale scores and total score of the ABQ should be used for Chinese elite athletes. However, direct comparisons on the scores derived from the Simplified Chinese version of ABQ between Chinese collegiate athletes and elite athletes should be cautioned.

## Data Availability Statement

The raw data supporting for the conclusions of this study are available from the corresponding author on reasonable request.

## Ethics Statement

The studies involving human participants were reviewed and approved by the Hong Kong Baptist University. Written informed consent to participate in this study was provided by the participants’ legal guardian/next of kin.

## Author Contributions

HL, X-BJ, Z-QG, and J-DL: conceptualization. HL, X-BJ, D-HW, and J-DL: methodology. HL, Z-QG, and J-DL: validation. XW, R-HY, J-CH, and J-DL: formal analysis. X-BJ, J-CH, D-HW, HL, and Y-DZ: investigation. X-BJ, D-HW, J-DL, and Y-DZ: Resources. J-CH, XW, and R-HY: data curation and project administration. J-DL, HL, and XW: writing original draft preparation and review and editing. J-DL: supervision. All authors have read and approved the final manuscript.

## Conflict of Interest

The authors declare that the research was conducted in the absence of any commercial or financial relationships that could be construed as a potential conflict of interest.

## Publisher’s Note

All claims expressed in this article are solely those of the authors and do not necessarily represent those of their affiliated organizations, or those of the publisher, the editors and the reviewers. Any product that may be evaluated in this article, or claim that may be made by its manufacturer, is not guaranteed or endorsed by the publisher.
